# Global Research Trends in Tyrosine Kinase Inhibitors: Coword and Visualization Study

**DOI:** 10.2196/34548

**Published:** 2022-04-08

**Authors:** Jiming Hu, Kai Xing, Yan Zhang, Miao Liu, Zhiwei Wang

**Affiliations:** 1 School of Information Management Wuhan University Wuhan China; 2 Department of Cardiovascular Surgery Renmin Hospital of Wuhan University Wuhan China; 3 Department of Clinical Laboratory Renmin Hospital of Wuhan University Wuhan China; 4 Department of Pediatrics Renmin Hospital of Wuhan University Wuhan China

**Keywords:** TKIs, coword analysis, literature visualization, NSCLC, targeted therapy, CML, topics distribution, HER2, pharmacokinetics

## Abstract

**Background:**

Tyrosine kinase inhibitors (TKIs) have achieved revolutionary results in the treatment of a wide range of tumors, and many studies on this topic continue to be published every year. Some of the published reviews provide great value for us to understand TKIs. However, there is a lack of studies on the knowledge structure, bibliometric analysis, and visualization results in TKIs research.

**Objective:**

This paper aims to investigate the knowledge structure, hotspots, and trends of evolution of the TKIs research by co-word analysis and literature visualization and help researchers in this field to gain a comprehensive understanding of the current status and trends.

**Methods:**

We retrieved all academic papers about TKIs published between 2016 and 2020 from the Web of Science. By counting keywords from those papers, we generated the co-word networks by extracting the co-occurrence relationships between keywords, and then segmented communities to identify the subdirections of TKIs research by calculating the network metrics of the overall and local networks. We also mapped the association network topology, including the network within and between TKIs subdirections, to reveal the association and structure among varied subdirections. Furthermore, we detected keyword bursts by combining their burst weights and durations to reveal changes in the focus of TKIs research. Finally, evolution venation and strategic diagram were generated to reveal the trends of TKIs research.

**Results:**

We obtained 6782 unique words (total frequency 26,175) from 5584 paper titles. Finally, 296 high-frequency words were selected with a threshold of 10 after discussion, the total frequency of which accounted for 65.41% (17,120/26,175). The analysis of burst disciplines revealed a variable number of burst words of TKIs research every year, especially in 2019 and 2020, such as HER2, pyrotinib, next-generation sequencing, immunotherapy, ALK-TKI, ALK rearrangement. By network calculation, the TKIs co-word network was divided into 6 communities: C1 (non-small–cell lung cancer), C2 (targeted therapy), C3 (chronic myeloid leukemia), C4 (HER2), C5 (pharmacokinetics), and C6 (ALK). The venation diagram revealed several clear and continuous evolution trends, such as non-small–cell lung cancer venation, chronic myeloid leukemia venation, renal cell carcinoma venation, chronic lymphocytic leukemia venation. In the strategic diagram, C1 (non-small–cell lung cancer) was the core direction located in the first quadrant, C2 (targeted therapy) was exactly at the junction of the first and fourth quadrants, which meant that C2 was developing; and C3 (chronic myeloid leukemia), C4 (HER2), and C5 (pharmacokinetics) were all immature and located in the third quadrant.

**Conclusions:**

Using co-word analysis and literature visualization, we revealed the hotspots, knowledge structure, and trends of evolution of TKIs research between 2016 and 2020. TKIs research mainly focused on targeted therapies against varied tumors, particularly against non-small–cell lung cancer. The attention on chronic myeloid leukemia and pharmacokinetics was gradually decreasing, but the focus on HER2 and ALK was rapidly increasing. TKIs research had shown a clear development path: TKIs research was disease focused and revolved around “gene targets/targeted drugs/resistance mechanisms.” Our outcomes will provide sound and effective support to researchers, funders, policymakers, and clinicians.

## Introduction

### Background

Tyrosine kinases (TKs) are a collective term for dozens of kinases encoded by multiple genes, which can phosphorylate tyrosine residues in cells [1]. Based on varied cellular localizations, the TKs family is divided into receptor tyrosine kinases (RTKs) [2] and non-RTKs [3]. RTKs consist of 20 subfamilies (eg, epidermal growth factor receptor or EGFR [4], vascular endothelial growth factor receptor or VEGFR [5]), whereas non-RTKs include 10 subfamilies such as ABL, SRC, and CSK [6]. TKs have the common activity to catalyze the transfer of γ-phosphate groups on adenosine triphosphate to the tyrosine residues of a variety of target proteins [1,3-7], and this process plays a key role in signal transduction within the cell. Abnormal activities of TKs are closely associated with proliferation, invasion, metastasis, apoptosis, and tumor angiogenesis in non-small–cell lung cancer (NSCLC) [8], chronic myeloid leukemia (CML) [2,9], and many other tumors. Therefore, TKs have become excellent targets for tumor therapy.

Tyrosine kinase inhibitors (TKIs) are a class of small-molecule compounds that can specifically inhibit TKs. They can penetrate through the cell membrane and block the signaling pathway of tumor proliferation, with some TKIs also capable of inhibiting angiogenesis [1,10]. TKIs have revolutionized the treatment of a variety of tumors [10-12]; for example, imatinib has been a typical pioneer in successfully translating oncogene research into molecular targeted therapy. Now, TKIs have developed to the fourth generation, which aims to overcome drug resistance due to T790M and C797S mutations [13]. More than 30 small-molecule TKIs have been approved for marketing by the US Food and Drug Administration (FDA), and hundreds of drug candidates are in various stages of clinical trials [13-15]. Therefore, this article aims to understand the development process of TKIs research, identify the main research directions, and analyze the potential research hotspots.

Co-word analysis is a content analysis method to study the knowledge structure and evolutionary patterns of various fields. It can facilitate researchers to identify hotspots, composition, paradigms, and evolution of a field by calculating the word pairs and co-occurrence of noun phrases in the literature [16-18]. This method has been used widely in medical bibliometric analysis, such as precision medicine [16], neonatal ischemic-hypoxic encephalopathy [19], stem cell research [20], neural stem cells [17], tumor immunotherapy [18], disaster medicine [21], medical big data [22], surgical robotics [23], epilepsy genetics [24]. We propose to use the co-word analysis and literature visualization to explore the knowledge structure, evolution trends, and associations among subtopics of TKIs research, aiming to help clinicians and scholars have a comprehensive understanding of TKIs and to give suggestions for research and usage of TKIs.

### Literature Review

In recent years, targeted therapies have become a hotspot in the development of antitumor drugs with their advantages of high selectivity and low side effects [25,26]. TKIs are revolutionary targeted drugs that inhibit tumor proliferation by interfering with or inhibiting specific proteins within cancer cells, thus exerting prominent antitumor effects [1-3]. Among them, imatinib was the first targeted antitumor drug [27], which was first approved in 2001 for the treatment of BCR-ABL–positive and Philadelphia chromosome–positive CML [3,6]. And then, the first-, second-, and third-generation TKIs, represented by gefitinib, dasatinib, and osimertinib, have been validated in hundreds of clinical trials and approved for marketing [10-12,28,29].

Genetic testing has been developed rapidly. Next-generation sequencing allows for sequencing genome and exome within days and makes it possible to identify patients with druggable mutations quickly and precisely [30]. Meanwhile, multidisciplinary collaboration between pharmacology and clinical science has brought a leap forward in basic research and clinical applications of TKIs. First, tumor-targeted therapies are the most established area for TKIs, especially in the treatment of lung cancer [12,15,31,32] and leukemia [10,11,28,29]. TKIs have improved the quality of life and extended survival in patients with advanced NSCLCs [33]. Imatinib and gefitinib have become first-line drugs due to their outstanding clinical efficacy in patients with BCR-ABL–positive CML [10,11,28,29]. Second, clinical trials of various drugs targeting HER2 and ALK (eg, trastuzumab [34,35], palivizumab [36], ceritinib [37]) have manifested excellent effects. Third, pharmacokinetics is another focus of TKIs research. Optimization and selectivity study is an important direction for continuing clinical trials after the launch of many TKIs. Besides, individualized blood concentration monitoring is important for patients with poor efficacy or severe side effects [38].

### Previous Efforts

In recent years, TKIs have been widely used for tumor-targeted therapies. Numerous research efforts helped clinicians and scholars better understand TKIs and facilitated the clinical translation of study outcomes.

Based on recent reviews, the current status of TKIs research is summarized as follows: First, resistance to TKIs is becoming increasingly prominent, of which genetic mutations (eg, T790M [39,40], C797S [13], D761Y [41], L747S [42]) are the main cause. It has become essential to find new molecular mechanisms underlying resistance to TKIs and to establish individualized dosing regimens. Second, the application of drugs such as erlotinib [43], osimertinib [15,33], and gefitinib [44] has gradually matured and occupied an important position in the treatment of various tumors such as NSCLCs [11,12]. Third, drugs targeting HER2 and ALK continue to emerge, which offers new hope for solving the plague of drug resistance [45,46]. To date, hundreds of new TKIs candidates are in various stages of clinical research [47].

### Rationale for the Study

Research on TKIs continues to grow to benefit more patients. However, there is still a lot of uncharted territories to explore in TKIs research. How to discover new biomarkers of TKIs? How many new applications of TKIs have been discovered? How to select TKIs with better clinical effects and fewer side effects for targeted therapy? How to overcome multidrug resistance in patients with tumors? How to individualize the use of TKIs in precision medicine? All these questions need scientific bibliometric analysis based on the results of TKIs research. The purpose of our study is to address the following questions:

1. What is the overall knowledge structure of TKIs research?
2. What are the subdirections of TKIs research and how do they interact with each other?
3. What are the evolutionary status and development trends of TKIs research in the temporal dimension?

## Methods

### Data Collection and Processing

It is well known that Web of Science Core Collection (WOSCC) is the most extensive and comprehensive academic literature database, so we used keywords including “Tyrosine kinase inhibitor, Tyrosine kinase inhibitors, TKI, TKIs, Tyrosine kinases inhibitors, Tyrosine kinases inhibitors” in WOSCC to precisely search all studies about TKIs by limiting the period to 2016-2020 and the literature types to journal papers, reviews, and conference papers. The specific search formula was “(TS=(‘Tyrosine kinase inhibitor’ OR ‘Tyrosine kinase inhibitors’ OR ‘TKI’ OR ‘TKIs’ OR ‘Tyrosine kinases inhibitors’ OR ‘Tyrosine kinases inhibitor’)) AND LANGUAGE: (English) Refined by: DOCUMENT TYPES: ( ARTICLE OR REVIEW OR PROCEEDINGS PAPER ) Timespan: 2016-2020. Indexes: SCI-EXPANDED, SSCI, A&HCI, CPCI-S, CPCI-SSH, ESCI, CCR-EXPANDED, IC.”

**Figure 1:**
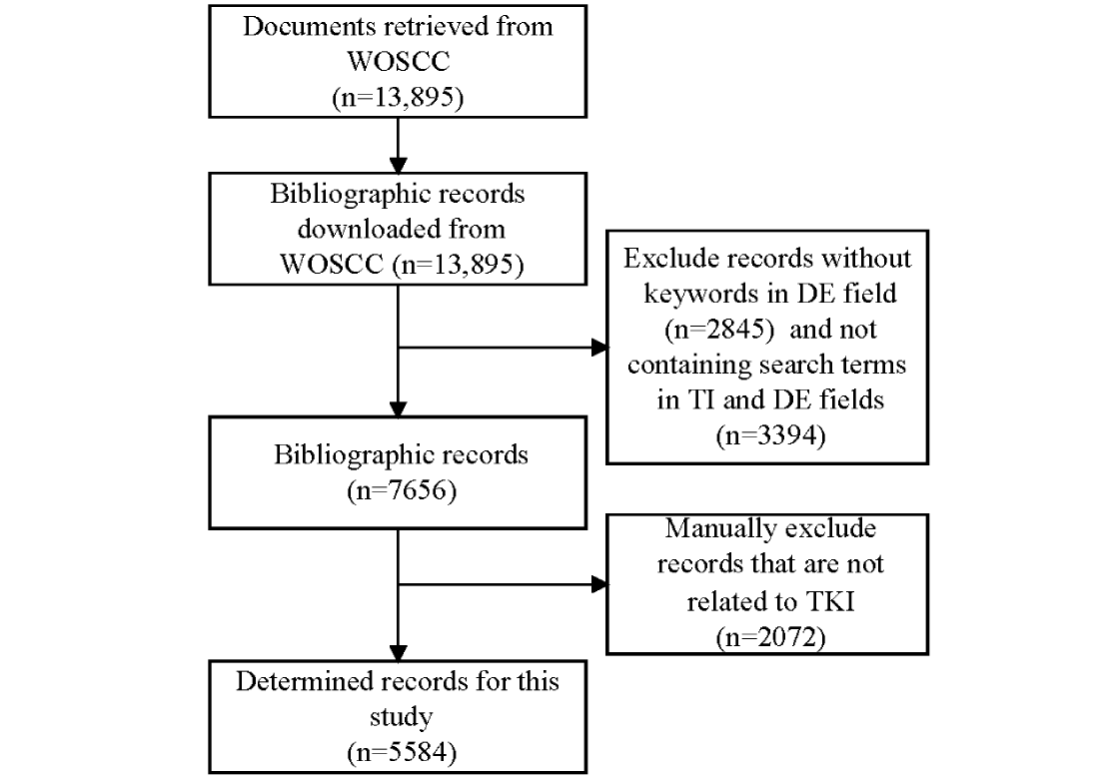
Search procedure for documents in TKIs research. DE: descriptor; TKI: tyrosine kinase inhibitor; TI: title; WOSCC: Web of Science Core Collection.

A total of 13,895 documents were retrieved and exported in the tab-delimited (Win) format. Next, the records containing the aforesaid search terms in the titles or keywords were retained, while those without keywords and with search terms appearing only in the abstracts were excluded [16,48]. Finally, 5584 records were obtained for the subsequent co-word network analysis (Figure 1).

Because there are irregularities and inconsistencies in the writing of keywords submitted by the authors themselves in WOS, it is necessary to preprocess them. First, this paper aims to depict the research status of TKIs by using other terms associated with TKIs, so TKI itself as well as the synonyms and hypernyms of TKI were removed. Keywords whose meaning is broad (eg, review, development, problem) were also removed. Second, the co-word analysis generally targets high-frequency keywords and their relationships, as keywords with very low frequencies cannot reflect the main direction of this research. Therefore, this paper (1) generated a list of keywords by frequency-descending order and (2) then defined the threshold of high-frequency keywords according to the cumulative percentage of frequencies in the list [48]. In the next step, (3) keywords with frequencies below the threshold were merged into the words with the closest meanings; besides, words with the same meaning but different forms were merged, such as “BCR-ABL TKI” to “BCR-ABL” and “epidermal growth factor receptor” to “EGFR.” Finally, (4) after deduplicating the merged keywords, a new list of keyword frequencies was generated.

### Network Construction and Analysis

The keywords in a paper are an accurate description of its main content, so mining keywords and their relationships can help reveal the hidden connotation of a research field [49]. If 2 words co-occur in the same connotation unit (eg, keywords in a paper), they are related or similar in connotation and have consistency in connotation expression. Their co-occurrence frequency is equal to the number of papers that contain them at the same time, and the greater the frequency, the stronger the semantic association between them [50]. By constructing co-word networks and performing structural analysis and visualization, co-word analysis can effectively reveal the underlying connotations, research structures, and even evolutionary trends of a research field [51].

In this paper, the above preprocessed data were imported into SCI2 [52] for frequency statistics and co-word network generation (.net format). Then, the .net file was imported into the network analysis tool Pajek [53] to calculate network indicators, including centralization and centrality [54], density [55], and the clustering coefficient [56], and to perform community segmentation to identify major subdirections. Centralization refers to the centripetal or consistency of the co-word network as a whole, while centrality reflects the keywords’ position in the network and their ability to influence and control the network [54]. Density represents the degree of association of the network as a whole, and the stronger the association, the more mature the research field. The clustering coefficient reflects the possibility that words will cluster into classes depending on the association and its strength, and the possibility that the network will be distinctly divided into several subnetworks or subclasses. Combined with the community segmentation algorithm (Louvain) [57], the co-word network will be divided into distinctive communities, each of which represents a subdirection, with strong ties within the communities and loose ties between the communities, reflecting a greater concentration or consistency in the connotative associations of words within the community. Keywords are tightly linked within communities and loosely linked between communities, reflecting that keywords possess more focused or consistent connotations within communities.

### Mapping and Visualization

To show the structure and characteristics of the TKIs research more intuitively and clearly, we visualized the topology, evolutionary venations, and development trend of the co-word network.

First, the visualization of the network topology was performed. VOSviewer [58] was used for the multilevel presentation of co-word networks and communities, including the intercommunity and intracommunity association network graphs. In the network graphs, nodes represent keywords or communities, and edges represent co-occurrence relationships between words or communities. The size of nodes and the thickness of lines are proportional to the frequency of keywords and the scale of communities, respectively, and the nodes and lines belonging to different communities are distinguished by different colors. These network diagrams visualize the importance and association relationships of keywords or communities in TKIs and help to analyze the distribution and structural characteristics of TKIs research.

Second, the visualization of evolutionary venations was performed. We divided each year’s records into several communities. Then we used Cortext [59] to calculate the overlapping relationships between communities in adjacent years and connected them through “tubes.” In the tube diagram, bars of different colors and sizes represent communities of different sizes, and the tubes connected by several bars represent the continuation of the research theme, which can be considered as evolutionary venations. The evolutionary trends of TKIs research over time are visualized by graphically characterizing the continuity, convergence, and divergence of communities.

Third, the visualization of the developmentary degree of the subdirections of TKIs research was performed. These research communities can be considered as subdirections of TKIs research, and each community or subdirection exhibits specific development status depending on the density and centrality. So, we drew a 2D strategic diagram based on the calculation of the density and centrality of each community. The strategic diagram took centrality, which represented the core degree of research directions in TKIs, as the horizontal axis, and density, which represented the developmental maturity of research directions, as the vertical axis, and the mean of community density and centrality as the origin. Ultimately, communities were mapped into 4 quadrants to visualize the degree of centrality and maturity of different research directions in TKIs.

Fourth, the visualization of burst words was performed. The changes in keyword frequency fluctuate significantly, with some of the words appearing in sudden bursts, reflecting the existence of distinct epochal characteristics of TKIs research. Therefore, we detected keyword bursts and combined their burst weights and durations to reveal changes in the focus of TKIs research [60].

## Results

### Themes Involved in TKIs Research

**Figure 2:**
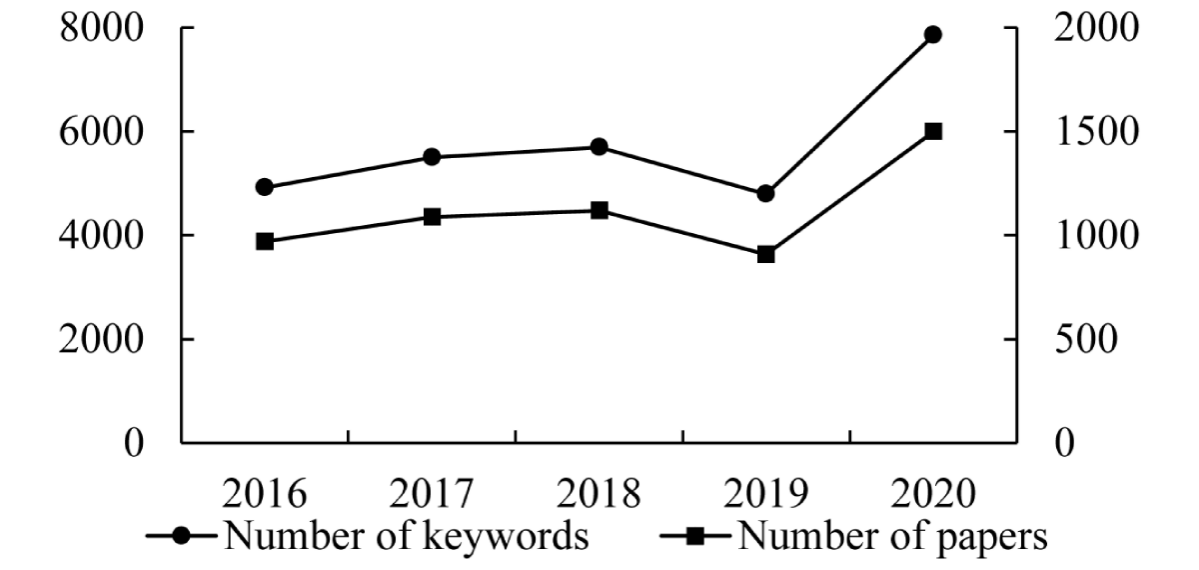
Yearly number of papers and words related to tyrosine kinase inhibitor (TKI) research (2016-2020).

We extracted 10,956 unique keywords from the 5584 available paper titles, and their total frequency was 28,743 (Figure 2). After preprocessing, 6782 unique words with a total frequency of 26,175 were left. After several rounds of testing and discussion, the threshold value of high-frequency words was taken as 10 in this paper. So after merging the keywords with frequencies lower than 10 into their superordinate words, we finally obtained 296 keywords for the subsequent co-word analysis (Table 1 and Multimedia Appendix 1). These 296 keywords, whose total frequency accounted for 65.41% (17,120/26,175), can represent the mainstream of TKIs research in the past 5 years and can also reflect a strong concentration trend of TKIs research.

**Figure 3:**
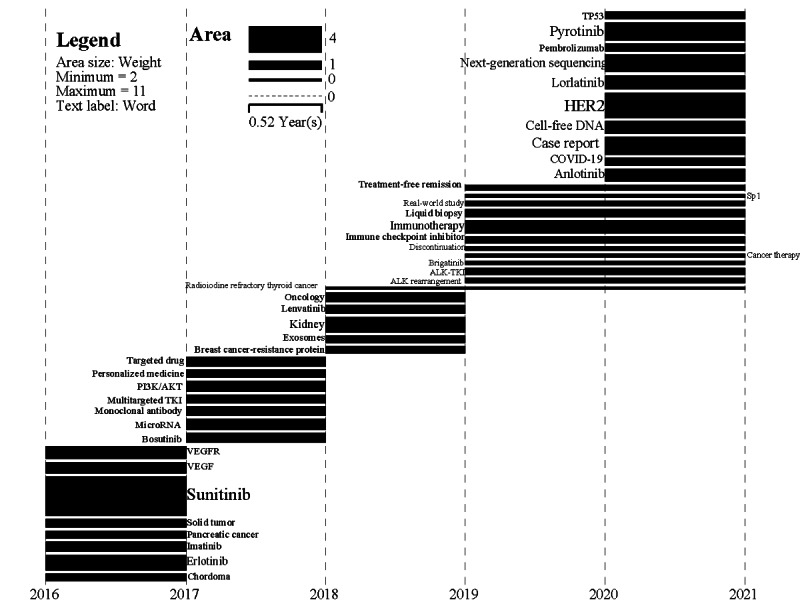
Burst disciplines of TKI research from 2016 to 2021. TKI: tyrosine kinase inhibitor; VEGFR: vascular endothelial growth factor receptor.

Burst keywords can represent important changes in TKIs research. Figure 3 shows a varying number of burst words in TKIs research each year, whose duration is expressed in terms of the length of the horizontal bar and weight in terms of the area. As can be seen from Figure 3, a variable number of emergent terms have appeared in TKIs research every year since 2016, especially in 2019 and 2020, indicating the emergence of new research themes in this field every year. The greater weight of burst words in 2020-2021 (eg, HER2, pyrotinib, next-generation sequencing, COVID-19, immunotherapy, ALK-TKI, ALK rearrangement, cell-free DNA, liquid biopsy, personalized medicine) suggests that these words in TKIs research were extensively explored by researchers in 2020.

**Table 1:** Top 30 keywords in papers related to tyrosine kinase inhibitor (TKI) research.

Ranking	Words	Frequency
1	“Non-small cell lung cancer”	1344
2	“EGFR”	916
3	“Chronic myeloid leukemia”	586
4	“EGFR-TKI”	506
5	“EGFR mutation”	404
6	“Lung cancer”	370
7	“Erlotinib”	299
8	“Imatinib”	283
9	“Osimertinib”	261
10	“Gefitinib”	257
11	“Targeted therapy”	256
12	“Renal cell carcinoma”	227
13	“Sunitinib”	219
14	“Lung adenocarcinoma”	207
15	“Mutation”	201
16	“Resistance”	192
17	“Afatinib”	186
18	“Chemotherapy”	183
19	“Cancer”	175
20	“Dasatinib”	162
21	“Drug resistance”	159
22	“T790M”	156
23	“ALK”	156
24	“Brain metastasis”	136
25	“Tumor”	134
26	“BCR-ABL”	128
27	“Nilotinib”	121
28	“Crizotinib”	120
29	“HER2”	119
30	“Apoptosis”	113

### Correlation Structure of Keywords in TKIs Research

#### Overview

Our analysis revealed that the co-word network consisting of the 296 keywords was exactly the maximal connected subgraph, that is, none of the high-frequency words in TKIs research is isolated, and all of them have paths associated with others, indicating that the research topics in this field form a whole that is interrelated and interact with each other.

#### Indicators of the Correlation Network

The overall indicators of the co-word network are shown in Table 2. The average degree of the network is 49.58, which means that a keyword in TKIs research is directly associated with 49.58 other keywords on average. These 49.58 keywords represent 16.75% of the entire network, which is a relatively small percentage, indicating that the range of intertopic associations in TKIs research is not extensive. The high degree of centralization of the network indicates a strong tendency to be centripetal or concentrated; the high closeness centralization and low betweenness centralization indicate that keywords are most directly related to each other rather than indirectly related; the high clustering coefficient indicates that keywords are likely to cluster into communities with certain words as the core. Collectively, TKIs research has clustered in certain specific subdirections in recent years, between which the distinction is obvious. However, the density of the current co-word network is not high, that is, the keywords are not closely related to each other, which indicates that TKIs research is more seriously fragmented and does not form a unified and mature research identity. We further divided the TKIs co-word network into 6 communities and calculated the module degree [61] to ensure a good division.

The network indicators of the keywords reflected their position and role in the TKIs co-word network (Table 3). Non-small cell lung cancer, EGFR, targeted therapy, lung cancer, EGFR-TKI, erlotinib, chemotherapy, cancer, sunitinib, and resistance all appear in the top 10 list of degree centralization and closeness centralization. The research topics associated with these words play an important role and have a strong influence on the whole field, while other words are likely to be clustered into a community with the above words as the core, forming a distinctive research subdirection. In addition to resistance, the above words also appear in the top 10 list of betweenness centralization, which serves as “bridges” in TKIs research, suggesting that more collaborations or synergies between TKIs research need to pass through these terms.

**Table 2:** The whole network indicators.

Indicators	Value
Number of nodes	296
Number of lines	7338
Average degree	49.5811
Network all degree centralization	0.5953
Network all closeness centralization	0.5265
Network betweenness centralization	0.0525
Network clustering coefficient	0.4702
Density	0.1681
Number of communities	6 (Modularity: 0.3062)

**Table 3:** Top 10 keywords in terms of degree, betweenness, and closeness centrality.

Ranking	Words	Degree	Words	Closeness	Words	Betweenness
1	“Non-small cell lung cancer”	224	“Non-small cell lung cancer”	0.8060	“Non-small cell lung cancer”	0.0552
2	“EGFR”	204	“EGFR”	0.7642	“EGFR”	0.0397
3	“Targeted therapy”	180	“Targeted therapy”	0.7195	“Targeted therapy”	0.0346
4	“Lung cancer”	179	“Lung cancer”	0.7178	“Chemotherapy”	0.0320
5	“EGFR-TKI”	166	“EGFR-TKI”	0.6958	“Lung cancer”	0.0291
6	“Erlotinib”	164	“Erlotinib”	0.6925	“EGFR-TKI”	0.0251
7	“Chemotherapy”	161	“Chemotherapy”	0.6876	“Erlotinib”	0.0216
8	“Cancer”	153	“Cancer”	0.6751	“Sunitinib”	0.0214
9	“Sunitinib”	151	“Sunitinib”	0.6720	“Imatinib”	0.0211
10	“Resistance”	142	“Resistance”	0.6585	“Cancer”	0.0206

#### Analysis of Thematic Communities

Depending on the association between keywords, TKIs are divided into 6 clusters or communities with certain important terms at their core. They are C1 (NSCLC); C2, targeted therapy; C3, CML; C4, HER2; C5, pharmacokinetics; and C6, ALK (Table 4). These 6 communities represent the subdirections of TKIs research in 2016-2020. They are each closely associated within but loosely associated with each other.

In terms of size, the subdirections of TKIs research can be divided into 3 echelons (Table 5). The first echelon includes C1 (NSCLC), which contains EGFR, EGFR-TKI, EGFR mutation, lung cancer, erlotinib, etc.; and C2 (targeted therapy), which contains renal cell carcinoma, sunitinib, chemotherapy, cancer, tumor, etc. These 2 major subdirections contain the largest number of keywords and the largest sum of frequencies, which are the main subdirections of TKIs research. The second echelon includes C3 (CML), which contains imatinib, dasatinib, BCR-ABL, nilotinib, gastrointestinal stromal tumor, etc.; C4 (HER2), which contains apoptosis, breast cancer, lapatinib, autophagy, combination therapy, etc.; and C5 (pharmacokinetics), which contains ibrutinib, metabolism, molecular docking, plasma, drug-drug interaction, etc. These 3 communities are involved in research topics that are also important for TKIs research. Keywords from these 3 communities are also important themes in TKIs research. The third echelon only contains C6 (ALK), including crizotinib, Met, RTK, ROS1, and ALK-TKI. C6 is still in its infancy, which occupies only a little weight in TKI research.

The varying centrality and density of each community further illustrate that there are sharply differentiated subdirections in TKIs research (Table 5). For example, C1 (NSCLC) and C2 (targeted therapy) have the highest centrality, which are the core subdirections in TKIs research. Besides, C1 (NSCLC) and C2 (targeted therapy) have the highest density and are the most developed subdirections in TKI research. Furthermore, the internal density of each community is higher than the density of the whole TKIs co-word network, which also indicates that each subdirection is tightly connected internally but loosely connected to each other. The centrality and density of C6 (ALK) do not have comparative value due to its small size.

**Table 4:** Topic communities related to tyrosine kinase inhibitor (TKI) research.

Community	Words^a^
C1-64	“Non-small cell lung cancer”; “EGFR”; “EGFR-TKI”; “EGFR mutation”; “lung cancer”; “erlotinib”; “osimertinib”; “gefitinib”; “lung adenocarcinoma”; “mutation”; “resistance”; “afatinib”; “drug resistance”; “T790M”; “brain metastasis”; “acquired resistance”; “next-generation sequencing”; “adenocarcinoma”; “T790M mutation”; “circulating tumor DNA”; “icotinib”; “liquid biopsy”; “epithelial-mesenchymal transition”; “EGFR-TKI resistance”; “sequencing”; “small cell lung cancer”; “case report”; “bevacizumab”; “gefitinib resistance”; “pemetrexed”; “squamous cell carcinoma”; “KRAS”; “epidermal growth factor”; “real-world study”; “leptomeningeal metastasis”; “advanced NSCLC”; “cost-effectiveness”; “rebiopsy”; “dacomitinib”; “IGF-1R”; “STAT3”; “droplet digital PCR”; “whole-brain radiotherapy”; “pleural effusion”; “BIM”; “uncommon mutation”; “polymorphism”; “Met amplification”; “first-line treatment”; “EGFR exon 20”; “computed tomography”; “cell-free DNA”; “TP53”; “radiosurgery”; “cisplatin; “docetaxel”; “leptomeningeal carcinomatosis”; “skin rash; “exosomes”; “cerebrospinal fluid”; “exon 19 deletion”; “exon 19”; “cetuximab”; “metformin”
C2-97	“Targeted therapy”; “renal cell carcinoma”; “sunitinib”; “chemotherapy”; “cancer”; tumor”; “metastasis”; “sorafenib”; “prognosis”; “pazopanib”; “hepatocellular carcinoma”; “lenvatinib”; “apatinib”; “immunotherapy”; “toxicity”; “metastatic renal cell carcinoma”; “radiotherapy”; “VEGF”; “progression-free survival”; “biomarker”; “angiogenesis”; “overall survival”; “adverse event”; “carcinoma”; “meta-analysis”; “sarcoma”; “VEGFR2”; “oncology”; “cabozantinib”; “renal cancer”; “VEGFR-TKI”; “PD-L1”; “VEGFR”; “clinical trial”; “axitinib”; “immune checkpoint inhibitor”; “molecular targeted therapy”; “thyroid cancer”; “FGFR”; “PDGFR”; “Phase I clinical trial”; “cardiotoxicity”; “vandetanib”; “regorafenib”; “angiogenesis inhibitor”; “PD-1”; “ovarian cancer”; “colorectal cancer”; “hypertension”; “anlotinib”; “bone metastasis”; “microRNA”; “recurrence”; “soft tissue sarcoma”; “mTOR”; “hypoxia”; “anti-angiogenesis”; “nivolumab”; “prognostic factor”; “AXL”; “RET”; “phase II clinical trial”; “everolimus”; “melanoma”; “anaplastic thyroid cancer”; “differentiated thyroid cancer”; “receptor TKI”; “clear cell renal cell carcinoma”; “medullary thyroid cancer”; “cancer therapy”; “mTOR inhibitor”; “kidney”; “adjuvant”; “tumor microenvironment”; “solid tumor”; “treatment response”; “multitargeted TKI”; “neutrophil-lymphocyte ratio”; “toceranib”; “multikinase inhibitor”; “antiangiogenic therapy”; “monoclonal antibody”; “sequential treatment”; “osteosarcoma”; “neoplasm metastasis”; “tolerability”; “esophageal cancer”; “hypothyroidism”; “circulating tumor cell”; “neoadjuvant therapy”; “Met TKI”; “PDGF”; “paclitaxel”; “neuroblastoma”; “oligoprogression”; “cervical cancer”; “pembrolizumab”
C3-46	“chronic myeloid leukemia”; “imatinib; dasatinib”; “BCR-ABL”; “nilotinib”; “gastrointestinal stromal tumor”; “acute myeloid leukemia”; “leukemia”; “molecular response”; “ponatinib”; “acute lymphoblastic leukemia”; “Philadelphia chromosome”; “TKI resistance”; “FLT3”; “kit”; “quality of life”; “adherence”; “head and neck squamous cell carcinoma”; “imatinib resistance”; “stem cell transplantation”; “leukemia stem cell”; “bosutinib”; “stem cell”; “Ph^+^ALL”; “treatment-free remission”; “protein kinase inhibitor”; “pulmonary arterial hypertension”; “minimal residual disease”; “PDGFRA”; “discontinuation”; “T315I”; “adverse drug reaction”; “chronic phase”; “cytokine”; “interferon”; “c-Kit”; “midostaurin”; “single nucleotide polymorphism”; “ruxolitinib”; “BCR-ABL mutation”; “kit mutation”; “treatment discontinuation”; “rechallenge”; “Src tyrosine kinase”; “BCR-ABL TKI”; “patient-reported outcome”
C4-38	“HER2”; “apoptosis”; “breast cancer”; “lapatinib”; “autophagy”; “combination therapy”; “neratinib”; “c-Met”; “gastric cancer”; “glioblastoma”; “AKT”; proliferation”; “nanoparticles”; “ERK”; “pancreatic cancer”; “reactive oxygen species”; “cancer stem cell”; “chemoresistance”; “Src”; “hepatotoxicity”; “oxidative stress”; “cell cycle”; “pyrotinib”; “radiation”; “PI3K”; “mitochondria”; “trastuzumab”; “migration”; “NF-kappa B”; “gemcitabine”; “drug delivery”; “glioma”; “triple-negative breast cancer”; “diarrhea”; “adjuvant therapy”; “metastatic breast cancer”; “PI3K/AKT”; “invasion”
C5-37	“Pharmacokinetics”; “ibrutinib”; “metabolism”; “molecular docking”; “plasma”; “drug-drug interaction”; “P-glycoprotein”; “therapeutic drug monitoring”; “Bruton tyrosine kinase”; “BTK inhibitor”; “nintedanib”; “chronic lymphocytic leukemia”; “positron emission tomography”; “LC-MS/MS”; “personalized medicine”; “anticancer”; “lymphoma”; “breast cancer resistance protein”; “interstitial lung disease”; “spleen tyrosine kinase”; “multidrug resistance”; “molecular dynamics”; “antitumor”; “lung”; “inflammation”; “diabetes”; “anticancer drug”; “BCL-2”; “bioavailability”; “synthesis”; “human plasma”; “mantle cell lymphoma”; “idiopathic pulmonary fibrosis”; “virtual screening”; “UPLC-MS/MS”; “pulmonary fibrosis”; “blood-brain barrier”
C6-14	“ALK”; “crizotinib”; “Met”; “receptor tyrosine kinase”; “ROS1”; “ALK-TKI”; “alectinib”; “ALK rearrangement”; “immunohistochemistry”; “lorlatinib”; “BRAF”; “ceritinib”; “resistance mutation”; “brigatinib”

^a^Keywords in each community are listed in descending order of frequency.

**Table 5:** Indicators of 6 theme communities in tyrosine kinase inhibitor (TKI) research.

Community	Number of nodes	Number of lines	Total frequency	Average degree	Density
C1: Non-small–cell lung cancer	64	817	7106	61.8438	0.3989
C2: Targeted therapy	97	1386	4621	54.9588	0.2946
C3: Chronic myeloid leukemia	46	297	2332	36.3696	0.2807
C4: HER2	38	203	1226	44.1579	0.2812
C5: Pharmacokinetics	37	161	1230	35.8649	0.2352
C6: ALK	14	59	605	50.6429	0.6020

#### Visualization of the Correlation Network

**Figure 4:**
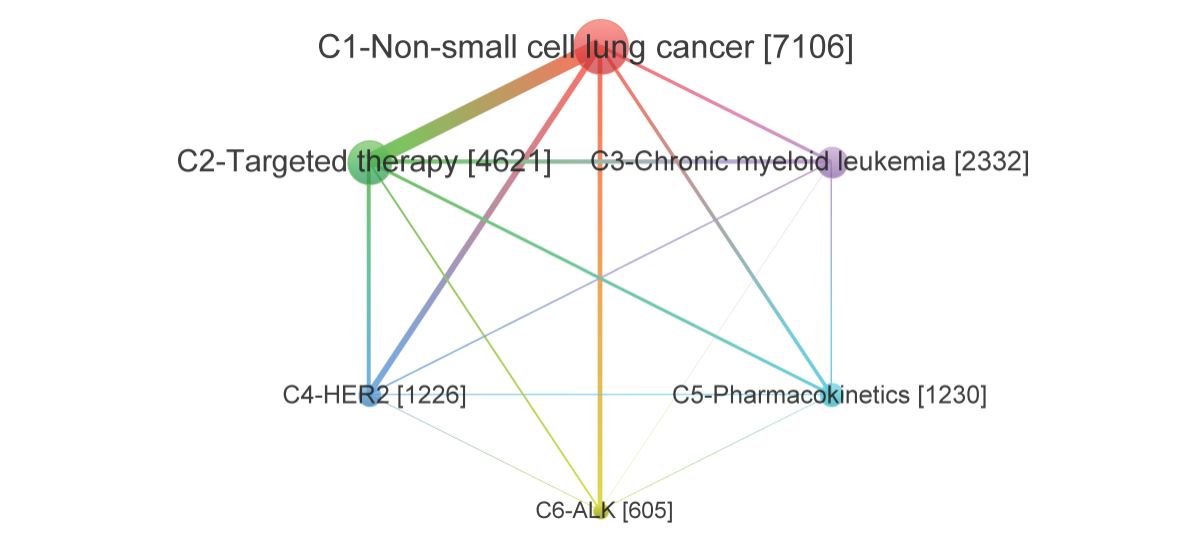
Correlation structure of subdirections in tyrosine kinase inhibitor (TKI) research.

**Figure 5:**
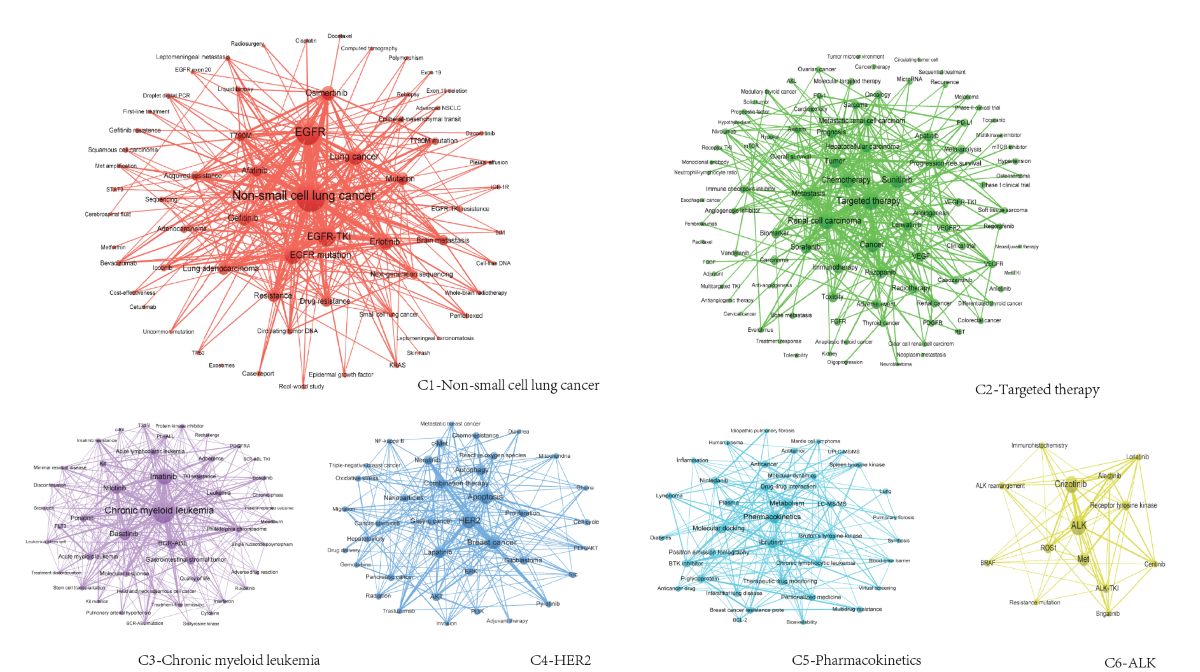
The internal correlation network structure of each subdirections in tyrosine kinase inhibitor (TKI) research.

The diverse correlation structure within and between each subdirection of the TKIs research is visualized in Figures 4 and 5. Figure 4 shows that there are differences in the influence of varied subdirections and that the association between these subdirections is uneven. C1 (NSCLC) and C2 (targeted therapy) have the strongest associations with the other subdirections, which reflect the strong influence of these 2 directions in TKIs research. The other four subdirections are oriented to C1 and C2, or depend on them to varying degrees. The association between C1 and C2 is significantly stronger than that between other subdirections, so C1 and C2 are the mainstream of current TKIs research. In particular, C1 is the most central and influential subdirection in the whole TKIs research, and the associations between C1 and other directions are generally strong. Isolated C6 (ALK) is not strongly associated with any other subdirection except for a closer association with C1.

Figure 5 further shows the correlation structure within each subdirection, where each term has a different location, function, and role. Each subdirection has a clear hierarchy, with the most influential terms at the core, which are important research themes, and the more distant from the core of the community, the less important the terms are. For example, EGFR, EGFR-TKI, EGFR mutation, lung cancer, and erlotinib are important research themes in C1 (NSCLC), which are closely related to the other terms or extended to other themes.

### Evolution Patterns of and Trends in TKIs Research

#### The Evolutionary Venations of Research Themes

**Figure 6:**
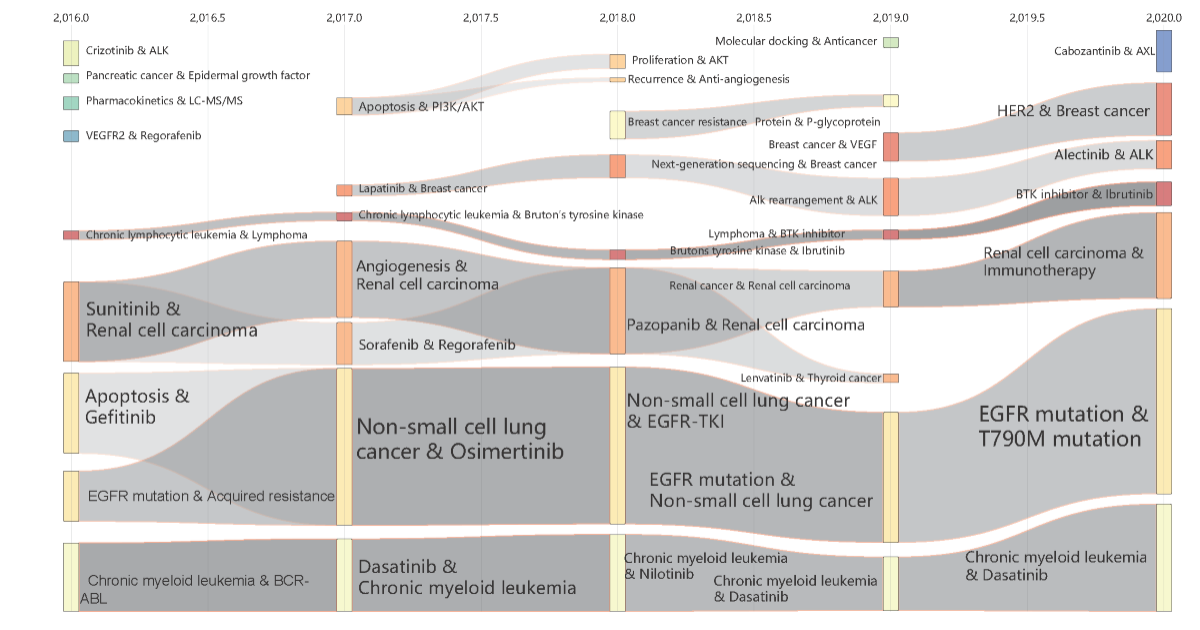
The evolution of themes of TKIs research over time (2016-2020). BTK: Bruton tyrosine kinase; EGFR: epidermal growth factor receptor; LC: liquid chromatography; MS/MS: tandem mass spectrometry; TKI: tyrosine kinase inhibitor; VEGFR: vascular endothelial growth factor receptor.

Figure 6 illustrates the historical evolution of TKIs research themes, which includes both the scale and the clear evolutionary venation of TKIs research themes. Overall, there are clear subdirections and good continuity of TKIs research from 2016 to 2020, but their size and distribution are uneven. In the descending order of size, several major evolutionary venations are the NSCLC venation (involving apoptosis, gefitinib, osimertinib, EGFR-TKI, T790M mutation, etc.), the CML venation (involving BCR-ABL, dasatinib, nilotinib, etc.), the renal cell carcinoma venation (involving sunitinib, angiogenesis, sorafenib, regorafenib, pazopanib, immunotherapy, etc.), the chronic lymphocytic leukemia venation (involving lymphoma, Bruton tyrosine kinase, ibrutinib, BTK inhibitor, etc.), and the lapatinib venation (involving breast cancer, next-generation sequencing, ALK rearrangement, ALK, alectinib, etc.). There are also isolated, intermittent research themes, such as crizotinib and ALK in 2016, apoptosis and PI3K/AKT in 2017, molecular docking and anticancer in 2019, and cabozantinib and AXL in 2020 for the first time as a subdirection.

#### The Developmentary Degree of the Subdirections of TKIs Research

**Figure 7:**
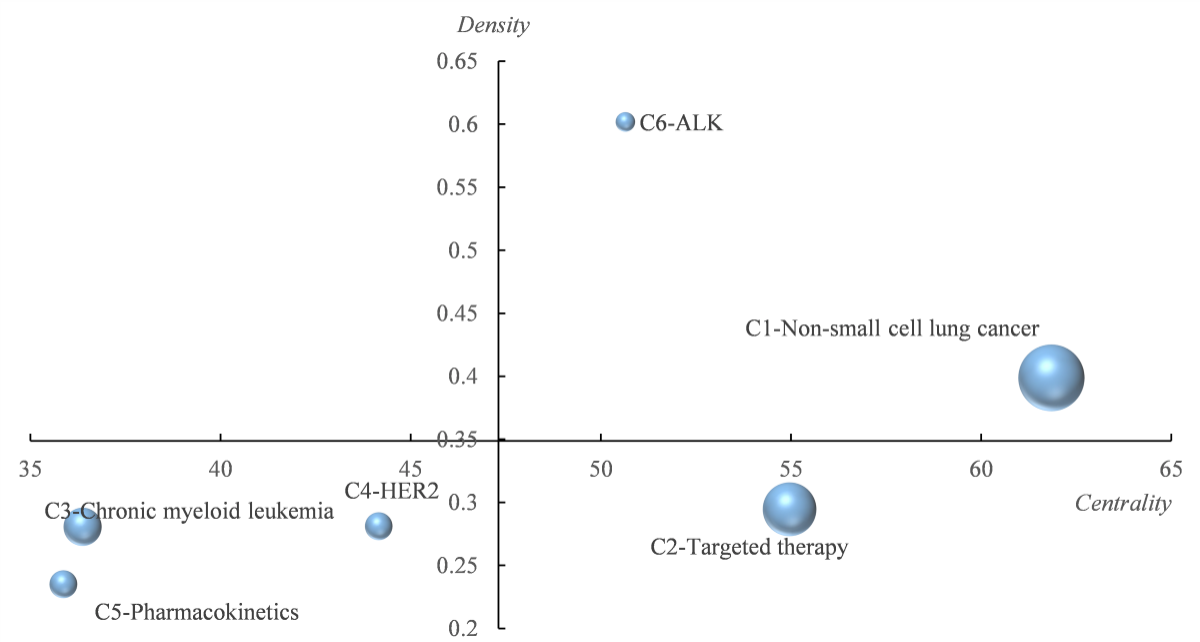
The relative development status and trends of 5 subdirections in the strategic diagram.

Based on Table 5, we drew a strategic diagram (Figure 7) to visualize the developmentary trends of the subdirections in TKIs research. The C6 community was not drawn in the strategy diagram due to its small size and noncomparable network indicators. As shown in Figure 7, we plotted the nodes of different sizes to represent the total frequencies of the varied subdirections of TKIs research and distributed them in 4 quadrants according to their density and centrality. C1 (NSCLC) is in the first quadrant due to its high density and centrality, again indicating that this community is the core direction and most developed in TKIs research. C2 (targeted therapy) is located exactly at the junction of the first and fourth quadrants, which means that C2 is also the core direction of TKIs research, but is in the process of maturing. C3 (CML), C4 (HER2), and C5 (pharmacokinetics) are all in the third quadrant, with relatively low centrality and density, which indicates that they are at the margins of TKIs research and are immature.

## Discussion

### Principal Findings

With the continuous progress of medical field, the targeted drugs (ie, TKIs) have made great development in basic research and have been widely used for clinical applications. Based on the results of the co-word analysis, we have a better understanding of the main research directions of TKIs and thus can accurately assess their maturity, centrality, and interactions. First, in general, TKIs research is unbalanced. The 296 words we selected from 6782 words accounted for 65.41% of the total word frequency and have a greater impact. Since the first TKIs were introduced, researchers have focused on some hot terms such as NSCLC, EGFR, CML, EGFR-TKI, EGFR mutation, erlotinib, imatinib, osimertinib, gefitinib, targeted therapy, renal cell carcinoma, resistance. These terms can be broadly classified into the following categories: clinical applications (eg, NSCLC, CML, renal cell carcinoma, lung adenocarcinoma), genetic studies (eg, EGFR, EGFR mutation, BCR-ABL), typical drugs (eg, erlotinib, imatinib, gefitinib, sunitinib, afatinib, dasatinib, osimertinib, nilotinib), and chemotherapy and drug resistance (eg, targeted therapy, resistance, chemotherapy, drug resistance). These terms not only reflect the areas of interest of researchers but also indicate the trends of TKIs research.

Based on the visualized co-word network, we found that the research topics tend to be clustered around a few keywords, eventually forming a hierarchical and relatively balanced thematic community. The thematic communities of the TKIs consist of C1 (NSCLC), C2 (targeted therapy), C3 (CML), C4 (HER2), C5 (pharmacokinetics), and C6 (ALK). There is also an imbalance between communities. First, the C1 and C2 communities are the main areas of TKIs research because of their high centrality and frequency. Among them, C1 (NSCLC), the largest thematic community, has received a lot of attention from scholars. From gene targets (eg, KRAS, EGFR, TP53, BIM) to signaling pathways (eg, IGF-1R, STAT3), from conventional chemotherapy (eg, cisplatin, docetaxel, pemetrexed) to targeted drugs (eg, erlotinib osimertinib, gefitinib), scholars have studied NSCLC in increasing depth. C2 (targeted therapy) indicates that TKIs are widely used in targeted therapies, and the application of TKIs has been extended to lung cancer [12,31,32], breast cancer [62], renal cancer [63,64], liver cancer [65], ovarian cancer [66], colorectal cancer [67], leukemia [11,28,29], thyroid cancer [68,69], cervical cancer [70], and many other tumors. Chemotherapy regimens containing TKIs were effective in reducing tumor metastasis and recurrence and improving the overall survival of patients [12,31]. The use of TKIs will be further expanded to more tumor types as more clinical trials are conducted.

Second, C3 and C5 communities have declined during the development of TKIs, both of which are in a marginal position in the strategic diagram. Among C3 (CML), imatinib is the first targeted antitumor drug that was first approved by the FDA in 2001 for patients with BCR-ABL–positive CML [10,11,28,29,71]. With the emergence of drug resistance, dasatinib [72], which targets the SRC, and nilotinib [73], which targets BCR-ABL, have been applied to patients who are resistant. Research on CML has been conducted for a long time and this field is now mature, so the application of TKIs has gradually expanded from CML to other diseases, which is leading to the gradual marginalization of the C3 community. C5 (pharmacokinetics) is an important interdisciplinary discipline related to TKIs, which is widely involved in the development process of TKIs [74,75]. However, as small-molecule drugs, the absorption, transport, distribution, and transformation of most TKIs in vivo have been clearly studied. Meanwhile, several new technologies in molecular biology (eg, molecular docking [76] and virtual high-throughput screening [77]) are used increasingly more, so C5 (pharmacokinetics) is gradually fading.

Despite the gradual decline of the C3 and C5 communities, new research areas such as C4 (HER) and C6 (ALK) have flourished in recent years. The overexpression, amplification, and mutations of HER2 have been found in a variety of tumors including breast cancer and NSCLC [45], and targeting HER2 has achieved excellent efficacy in breast cancer [62]. Although several early drugs targeting HER2 had poor efficacy in NSCLC [34,36,45], the advent of newer-generation HER2-targeting drugs such as poziotinib [78] and pyrotinib [79,80] exhibited good antitumor effects in clinical trials. Scholars are increasingly interested in targeting HER2 in NSCLC, while research on HER2 for breast cancer is relatively well established. Therefore, C4 may evolve in different directions in the future. The C6 (ALK) community is small but promising. ALK mutations, especially rearrangements, exhibit strong translational activity in NSCLC [81]. ALK-targeted agents such as alectinib [82] and brigatinib [83] have shown extraordinary efficacy in ALK-positive NSCLC and have become the first-line therapies [84]. Lorlatinib, an ALK inhibitor [85], appears as a burst word in 2020, and ALK-TKI and ALK rearrangement have a high weight from 2019 to 2020 (Figure 3), both of which indicate the rapidly rising attention on ALK. In addition, there are still several ALK-targeted drugs in development, so more literature on ALK will be published in the future and the C6 community will grow further.

We found several evolutionary venations by analyzing the evolution of themes of TKIs research over time. These highly concentrated evolutionary venations indicate scholars’ continuous and steady focus on NSCLC, CML, renal cell carcinoma, chronic lymphocytic leukemia, etc. These different evolutionary lines show a clear development path: TKIs research is disease focused and revolved around “gene targets/targeted drugs/resistance mechanisms.” For example, in the CML venation, investigators focused on the BRC-ABL in 2016 and on dasatinib and nilotinib in 2017-2020, which could both target BCR-ABL and overcome imatinib resistance [72,73]. In the NSCLC venation, investigators focused on EGFR genes in 2016, on EGFR-TKIs represented by osimertinib in 2017-2019, and on resistance mechanisms represented by T790M in 2020. In the renal cell carcinoma venation, investigators continued to focus on various TKIs such as sunitinib, sorafenib, regorafenib, and pazopanib, and also paid attention to gene targets such as PDGFR, FGFR, c-Kit, and VEGF, and multidrug resistance. In the chronic lymphocytic leukemia venation, investigators focused on B-cell–derived chronic lymphocytic leukemia and lymphoma in 2016, on the aberrant Bruton tyrosine kinase (BTK) from B cells in 2017, and on BTK inhibitors such as ibrutinib that can treat chronic lymphocytic leukemia and lymphoma in 2018-2020. Moreover, lapatinib, a dual EGFR/HER2 TKI [86], showed good efficacy in breast cancer, which made it an independent evolutionary venation in 2017 with continuous attention to date.

In addition to the main few evolutionary venations, we identified some isolated themes that depict the current state of TKIs research. Crizotinib gained attention as an ALK inhibitor in 2016, but its popularity declined rapidly due to its poor efficacy and the emergence of second-generation ALK inhibitors, making crizotinib and ALK an isolated topic. Circulating tumor DNA is important for efficacy assessment and prognosis analysis of tumors. The future trend in TKIs research will likely be to use next-generation sequencing or liquid biopsy technology to precisely analyze circulating tumor DNA in cell-free DNA. The PI3K/AKT pathway plays an important role in cell growth, proliferation, migration, and angiogenesis, which can be activated by RTKs. Thus, as one of the mechanisms of TKIs, the apoptosis and PI3K/AKT venation was noticed in 2017-2018. Cabozantinib is a multitarget TKI that can target 9 genes (eg, AXL, Met) [87], and cabozantinib and AXL appeared as a separate topic in 2020. Furthermore, the burst words pyrotinib and anlotinib, which accounted for a relatively large weight in 2020, are multitarget inhibitors [79,80,88]. This suggests that multitarget drugs may become an important direction for the development of TKIs and will likely receive more attention in the future. Immune checkpoint inhibitors and TKIs are important drugs for tumors. The combination of PD-L1 inhibitors (eg, pembrolizumab) and TKIs (eg, lenvatinib) in patients with malignant tumors was more effective than single drug [89], suggesting that the combination therapy was an important development direction for future tumor therapy. For well-known reasons, many patients being treated with TKIs were co-infected with SARS-CoV-2 in 2020 [90], and it was also suggested that some TKIs such as BTKs may have therapeutic effects on COVID-19 [91], which made COVID-19 a burst word in TKIs research. Because of global spread of COVID-19 in 2021, investigators’ interest in TKIs for patients infected with SARS-CoV-2 would further increase.

### Limitation

Our study also has some limitations: first, our search included only English literature from 2016 to 2020, while non-English literature was excluded; second, the co-word analysis did not take the quality, influence, and rigor of the literature into account, which was a common shortcoming of such papers [92-94].

### Conclusions

In conclusion, we presented a visualization of TKIs research during 2016-2020 utilizing co-word analysis and the hotspots, knowledge structure, and trends of evolution revealed in our work will help researchers in the field of TKIs to gain a comprehensive understanding of the current status and trends. Based on the above results, we speculate that the general status of TKIs research is as follows: (1) NSCLC and CML are the most important clinical application areas for TKIs; (2) EGFR is the most common target gene for TKIs, and EGFR-TKIs are the most commonly used molecularly targeted TKIs, among which erlotinib, osimertinib, and gefitinib have gradually matured; (3) TKIs have become a mature field for targeted therapeutic applications, and drugs targeting HER2 and ALK have further expanded the application of TKIs; and (4) drug resistance remains a major challenge for TKIs. In a nutshell, our work remains valuable in revealing the knowledge structure and evolutionary trends of TKIs research.

## Appendix

Multimedia Appendix 1 Top 100 keywords in papers related to research.

## Abbreviations

ATP: adenosine triphosphate
BTK: Bruton tyrosine kinase
ctDNA: circulating tumor DNA
CML: chronic myeloid leukemia
EGFR: epidermal growth factor receptor
FDA: Food and Drug Administration
NSCLC: non-small small–cell lung cancer
RTKs: receptor tyrosine kinases
TKIs: tyrosine kinase inhibitors
TKs: tyrosine kinases
VEGFR: vascular endothelial growth factor receptor
WOSCC: Web of Science Core Collection
