# Supplementary material for: Global Research Trends in Tyrosine Kinase Inhibitors: Coword and Visualization Study
Source: JMIR Med Inform. 2022 Apr 8;10(4):e34548. doi: 10.2196/34548 (PMC9034433; doi:10.2196/34548)
Supplement: Multimedia Appendix 1 [file medinform_v10i4e34548_app1.docx]

Supplementary table 1. Top 100 keywords in papers related to TKIs (tyrosine kinase inhibitor) research.

| **Ranking** | **Words** | **Frequency** | **Ranking** | **Words** | **Frequency** |
| --- | --- | --- | --- | --- | --- |
| 1 | Non-small Cell Lung Cancer | 1344 | 51 | Metabolism | 76 |
| 2 | EGFR | 916 | 52 | Angiogenesis | 73 |
| 3 | Chronic Myeloid Leukemia | 586 | 53 | Overall Survival | 72 |
| 4 | EGFR-TKI | 506 | 54 | Adenocarcinoma | 72 |
| 5 | EGFR Mutation | 404 | 55 | T790M Mutation | 71 |
| 6 | Lung Cancer | 370 | 56 | Met | 70 |
| 7 | Erlotinib | 299 | 57 | Lapatinib | 68 |
| 8 | Imatinib | 283 | 58 | Adverse Event | 68 |
| 9 | Osimertinib | 261 | 59 | Molecular Docking | 67 |
| 10 | Gefitinib | 257 | 60 | Carcinoma | 66 |
| 11 | Targeted Therapy | 256 | 61 | Meta-analysis | 64 |
| 12 | Renal Cell Carcinoma | 227 | 62 | Circulating Tumor DNA | 60 |
| 13 | Sunitinib | 219 | 63 | Autophagy | 59 |
| 14 | Lung Adenocarcinoma | 207 | 64 | Sarcoma | 58 |
| 15 | Mutation | 201 | 65 | Combination Therapy | 58 |
| 16 | Resistance | 192 | 66 | Acute Myeloid Leukemia | 58 |
| 17 | Afatinib | 186 | 67 | Icotinib | 58 |
| 18 | Chemotherapy | 183 | 68 | Leukemia | 55 |
| 19 | Cancer | 175 | 69 | VEGFR2 | 55 |
| 20 | Dasatinib | 162 | 70 | Oncology | 53 |
| 21 | Drug Resistance | 159 | 71 | Cabozantinib | 52 |
| 22 | T790M | 156 | 72 | Molecular Response | 52 |
| 23 | ALK | 156 | 73 | Ponatinib | 52 |
| 24 | Brain Metastasis | 136 | 74 | Liquid Biopsy | 51 |
| 25 | Tumor | 134 | 75 | Renal Cancer | 51 |
| 26 | BCR-ABL | 128 | 76 | Acute Lymphoblastic Leukemia | 50 |
| 27 | Nilotinib | 121 | 77 | Plasma | 48 |
| 28 | Crizotinib | 120 | 78 | Epithelial-mesenchymal Transition | 48 |
| 29 | HER2 | 119 | 79 | VEGFR-TKI | 48 |
| 30 | Apoptosis | 113 | 80 | Drug-drug Interaction | 47 |
| 31 | Metastasis | 111 | 81 | PD-L1 | 47 |
| 32 | Acquired Resistance | 111 | 82 | VEGFR | 47 |
| 33 | Sorafenib | 109 | 83 | Clinical Trial | 47 |
| 34 | Prognosis | 107 | 84 | EGFR-TKI Resistance | 46 |
| 35 | Breast Cancer | 107 | 85 | Axitinib | 46 |
| 36 | Pharmacokinetics | 107 | 86 | Philadelphia Chromosome | 45 |
| 37 | Pazopanib | 106 | 87 | P-glycoprotein | 45 |
| 38 | Next-generation Sequencing | 96 | 88 | TKI Resistance | 44 |
| 39 | Hepatocellular Carcinoma | 92 | 89 | Therapeutic Drug Monitoring | 44 |
| 40 | Gastrointestinal Stromal Tumor | 92 | 90 | Sequencing | 43 |
| 41 | Lenvatinib | 92 | 91 | Neratinib | 43 |
| 42 | Apatinib | 91 | 92 | Receptor Tyrosine Kinase | 43 |
| 43 | Immunotherapy | 91 | 93 | Bruton’s Tyrosine Kinase | 43 |
| 44 | Toxicity | 89 | 94 | Immune Checkpoint Inhibitor | 43 |
| 45 | Metastatic Renal Cell Carcinoma | 80 | 95 | Small Cell Lung Cancer | 42 |
| 46 | Radiotherapy | 80 | 96 | Molecular Targeted Therapy | 41 |
| 47 | VEGF | 78 | 97 | Thyroid Cancer | 41 |
| 48 | Ibrutinib | 78 | 98 | Case Report | 41 |
| 49 | Progression-free Survival | 77 | 99 | ROS1 | 40 |
| 50 | Biomarker | 76 | 100 | c-Met | 39 |
|  |  |  | 100-tie | FGFR | 39 |
|  |  |  | 100-tie | Bevacizumab | 39 |
|  |  |  | 100-tie | BTK Inhibitor | 39 |
